# Image-Based Modeling of Drug Delivery during Intraperitoneal Chemotherapy in a Heterogeneous Tumor Nodule

**DOI:** 10.3390/cancers15205069

**Published:** 2023-10-20

**Authors:** Mohsen Rezaeian, Hamidreza Heidari, Kaamran Raahemifar, Madjid Soltani

**Affiliations:** 1 Department of Mechanical Engineering, K. N. Toosi University of Technology, Tehran 19967-15433, Iran; mohsenrezaeian@email.kntu.ac.ir; 2 Otto H. York Department of Chemical and Materials Engineering, New Jersey Institute of Technology, University Heights, Newark, NJ 07102, USA; hh44@njit.edu; 3 Data Science and Artificial Intelligence Program, College of Information Sciences and Technology (IST), Penn State University, State College, PA 16801, USA; kvr5517@psu.edu; 4 School of Optometry and Vision Science, Faculty of Science, University of Waterloo, Waterloo, ON N2L 3G1, Canada; 5 Department of Chemical Engineering, Faculty of Engineering, University of Waterloo, Waterloo, ON N2L 3G1, Canada; 6 Department of Electrical and Computer Engineering, University of Waterloo, Waterloo, ON N2L 3G1, Canada; 7 Advanced Bioengineering Initiative Center, Multidisciplinary International Complex, K. N. Toosi University of Technology, Tehran 19967-15433, Iran; 8 Computational Medicine Center, K. N. Toosi University of Technology, Tehran 19967-15433, Iran; 9 Centre for Biotechnology and Bioengineering (CBB), University of Waterloo, Waterloo, ON N2L 3G1, Canada

**Keywords:** drug delivery, intraperitoneal chemotherapy, computational oncology, image-based spatiotemporal model, peritoneal carcinomatosis

## Abstract

**Simple Summary:**

Intraperitoneal (IP) chemotherapy is a treatment for cancers in the abdomen, delivering anti-cancer drugs directly into the peritoneal cavity. While this method has shown promising outcomes, limited drug penetration into tumors remains a challenge due to their unique pathophysiology. Our study aims to investigate drug delivery during IP chemotherapy using a mathematical model. We incorporated a real tumor image into our model to understand how tumor vessels and their distribution affect drug delivery. Our model allowed us to analyze the spatiotemporal distribution of drug concentration in the tumor. We also quantitatively evaluated treatment efficacy by examining drug availability in the tumor, drug penetration depth, and the fraction of killed cells during the treatment. Our findings revealed that each tumor’s specific vascular network can impact drug delivery during IP chemotherapy. Our model provides valuable insights into the challenges of IP chemotherapy and holds promise for applications in personalized medicine.

**Abstract:**

Intraperitoneal (IP) chemotherapy is a promising treatment approach for patients diagnosed with peritoneal carcinomatosis, allowing the direct delivery of therapeutic agents to the tumor site within the abdominal cavity. Nevertheless, limited drug penetration into the tumor remains a primary drawback of this method. The process of delivering drugs to the tumor entails numerous complications, primarily stemming from the specific pathophysiology of the tumor. Investigating drug delivery during IP chemotherapy and studying the parameters affecting it are challenging due to the limitations of experimental studies. In contrast, mathematical modeling, with its capabilities such as enabling single-parameter studies, and cost and time efficiency, emerges as a potent tool for this purpose. In this study, we developed a numerical model to investigate IP chemotherapy by incorporating an actual image of a tumor with heterogeneous vasculature. The tumor’s geometry is reconstructed using image processing techniques. The model also incorporates drug binding and uptake by cancer cells. After 60 min of IP treatment with Doxorubicin, the area under the curve (AUC) of the average free drug concentration versus time curve, serving as an indicator of drug availability to the tumor, reached 295.18 mol·m^−3^·s^−1^. Additionally, the half-width parameter W_1/2_, which reflects drug penetration into the tumor, ranged from 0.11 to 0.14 mm. Furthermore, the treatment resulted in a fraction of killed cells reaching 20.4% by the end of the procedure. Analyzing the spatial distribution of interstitial fluid velocity, pressure, and drug concentration in the tumor revealed that the heterogeneous distribution of tumor vasculature influences the drug delivery process. Our findings underscore the significance of considering the specific vascular network of a tumor when modeling intraperitoneal chemotherapy. The proposed methodology holds promise for application in patient-specific studies.

## 1. Introduction

Patients with malignancies within the peritoneal cavity are susceptible to metastasis in this region [1,2]. Peritoneal carcinomatosis (PC), also known as peritoneal dissemination, refers to the spread of malignancy along the lining surface of the peritoneal (abdominal) cavity [3]. PC stands as a grave consequence that menaces these patients, resulting in diminished quality of life and an unfavorable prognosis, attributed to issues like bowel obstructions and ascites [4,5,6]. While intravenous (IV) chemotherapy serves as a palliative measure for these cases, intraperitoneal (IP) chemotherapy, coupled with cytoreductive surgery (CRS), holds promise for treating these patients [7,8,9,10,11]. In IP administration, chemotherapy agents are introduced into the peritoneal cavity, directly exposing the tumor to the drug. Conversely, in IV injection, drug particles are transported to the tumor site through the blood circulation system and then penetrate the tumor by crossing the vessel wall. As a result, IP chemotherapy is considered a locoregional therapy that may provide improved efficacy with comparable systemic side effects to IV chemotherapy. Due to the direct drug–tumor contact following injection, this method yields a higher likelihood of drug penetration into the tumor. Nonetheless, drug penetration in IP chemotherapy remains limited, and this leads to poor efficacy of this treatment method [12]. Consequently, a comprehensive study of IP chemotherapy becomes imperative to thoroughly comprehend the factors constraining drug penetration within tumors.

Tumors have a unique pathophysiology. The extracellular matrix (ECM) in tumors is denser than in normal tissue [13,14,15]. Additionally, tumors lack an effective lymphatic system [16,17]. Tumor vessels are leakier than normal vessels, and their spatial distribution within the tumor is heterogeneous. Also, these vessels have irregular structures compared to healthy ones [18,19,20]. Collectively, these factors contribute to an elevated interstitial fluid pressure (IFP) within the tumor, while IFP sharply decreases at the tumor’s periphery [21]. This results in an outward convective flow at the tumor periphery, which impedes the convection-mediated penetration of drug particles, leaving diffusion as the primary mechanism for drug transport and penetration into the tumor. Nevertheless, the ECM structure in tumors further compromises the intratumoral diffusive transport of therapeutic agents [22]. In addition to these tissue-related factors, the ultimate distribution of the drug within the tumor is influenced by various therapy-related parameters, including dosage, temperature, the volume of the carrier fluid, intra-abdominal pressure, the potential use of vaso-active agents or surfactants, and treatment duration. Furthermore, the characteristics of the drug, such as its molecular weight, ionic charge, membrane binding, solubility, and diffusivity, also play a substantial role in determining how the drug distributes within the tumor [23]. To comprehend the impact of each of these factors on the drug transfer process, a comprehensive investigation encompassing the relevant parameters is essential. Mathematical modeling emerges as a potent tool, offering the capability to conduct cost-effective analyses. The in silico models of IP chemotherapy solve the governing equations related to interstitial fluid and mass transport, considering both the convection and diffusion mechanisms within the tumor. These models incorporate the characteristics of the tumor microenvironment, as well as the parameters associated with the drug and the treatment protocol. A mathematical investigation has the potential to offer deeper insights into the fundamental factors responsible for the limited drug penetration observed in IP chemotherapy. Furthermore, such a study could serve as a foundational platform for devising strategies aimed at enhancing the efficacy of this chemotherapy approach.

Until now, drug delivery to solid tumors has been studied in several works using mathematical models, with the majority of them focusing on IV chemotherapy [24,25,26,27,28,29,30,31,32,33]. One of the earliest models that concentrated on drug delivery during IP chemotherapy is the study by Au et al. [34], where a mathematical model was used to investigate the IP transfer of Paclitaxel to the tumor. In this model, spatially varying parameters were employed and the results were verified using a mouse model. Based on the framework first presented by Baxter and Jain [28,30,35] for intravascular injection of drugs into tumors, Steuperaert et al. [36] studied IP drug delivery in solid tumors accounting for diverse sizes and shapes of tumors. In this study, the concentration distribution in the tumor was investigated for two different drugs, including Paclitaxel and Cisplatin. The results of this model showed that IP injection works better for smaller tumors. Furthermore, the outcomes of this model indicated that vascular normalization therapy has the potential to elevate the depth of drug penetration into the tumor. Expanding upon this model in a study by the same group [37], a model was developed using DCE-MRI images of an actual mouse tumor to investigate the effect of the spatial distribution of the vascular network in the tumor. In this model, the tumor, normal tissue, and necrotic region were demarcated based on distinctive vascular properties derived from the real images of the tumor. The findings of this investigation revealed that the distribution of IFP in a heterogeneous tumor nodule is affected by the abnormal geometry and different segmentation of the tumor in terms of vascular properties.

Considering the shallow drug penetration depth within the tumor during IP chemotherapy as a basic limitation, Shamsi et al. [38] proposed IP delivery of magnetic nanoparticles to improve drug penetration. To achieve this objective, they examined the intraperitoneal transfer of drug-coated magnetic nanoparticles. The results of this study showed that using this technique can have a significant effect on increasing the penetration depth of the drug into solid tumors. Rezaeian et al. [39] proposed the use of a targeted drug delivery system using temperature-sensitive liposomes that releases encapsulated drug particle using the heat generated by high-intensity focused ultrasound. The results of this study showed that this two-stage drug delivery system can lead to increased drug penetration while also potentially mitigating the side effects associated with IP chemotherapy. Although the mentioned articles have investigated different aspects of IP chemotherapy, treating the tumor vasculature as a distributed source, none of the aforementioned studies explicitly addressed the heterogeneity of the tumor vasculature. Consequently, there is a noticeable gap in modeling studies that consider the actual tumor vasculature’s impact on drug distribution during IP chemotherapy. This aspect is particularly crucial, especially considering that vessels have been confirmed to function as localized drug sinks [40]. Our previous work in IV chemotherapy [32] demonstrated that image-based modeling can provide a more accurate representation of drug delivery to solid tumors. Consequently, it is anticipated that the heterogeneity of the tumor vasculature will also impact drug delivery during IP chemotherapy.

In this work, we modeled drug delivery during IP chemotherapy using a reconstructed geometry based on an actual image of a heterogeneous tumor vasculature. Using image processing techniques, tumor geometry is built based on a real image and considered as an input for our model. By solving the fluid flow and convection–reaction–diffusion equations, the transport of drugs within the tumor has been simulated. Moreover, the process of drug binding and uptake into cancer cells has been taken into account. Finally, by investigating IFV and IFP in the tumor, as well as the temporal and spatial distribution of drug concentration within the tumor, the performance of IP chemotherapy has been investigated. Also, the accuracy of the results has been compared and validated with previous studies.

## 2. Materials and Methods

**Figure 1:**
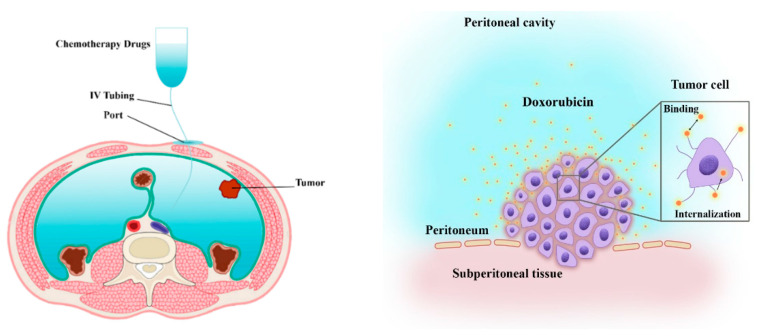
Schematic of drug delivery during IP administration of Doxorubicin. The IP chemotherapy is delivered to the patient’s abdomen using an IP port and catheter. Once the Doxorubicin particles enter the tumor interstitium, they can bind to cancer cells, unbind, or become internalized [39].

A schematic of chemotherapy with IP injection is shown in Figure 1. Therapeutic agents are administered intraperitoneally, where they can penetrate the tumor tissue. The diffusion and convection mechanisms aid in facilitating the deeper penetration of therapeutic agents within the tumor tissue [41]. Diffusive transport is determined by the concentration and diffusion coefficient of the drug within the interstitium. Convective transport, on the contrary side, is dependent on interstitial fluid velocity (IFV) and tissue permeability [16]. The drug can penetrate the tissue, attach or detach to receptors on the surface of cancerous cells, and finally internalize into the cells [25].

Darcy’s law for interstitial fluid flow, the laminar flow equation for intravascular flow, and convection–diffusion–reaction (CDR) equations for drug delivery in tumor tissue are all included in the mathematical model for IP chemotherapy. The diffusion and convection of therapeutic agents in the interstitium, as well as interactions of therapeutic agents and cancerous, such as binding/unbinding and internalization, are determined by the mass transport equations.

**Figure 2:**
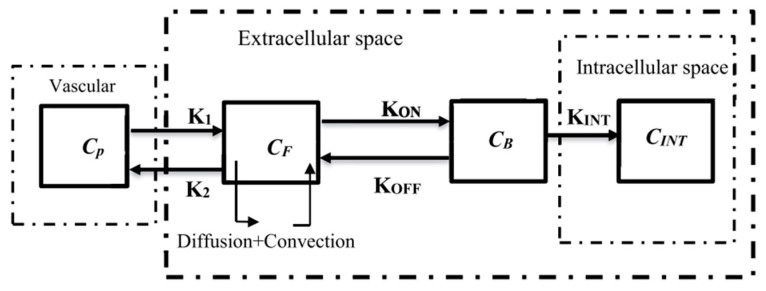
Compartmental model of IP Doxorubicin delivery in our model. CDR equations are added to the classic compartmental model [39].

The compartmental models, which are frequently employed to represent drug delivery, are the foundation of the generic mass transport model. The concentration of drug in each compartment is considered to be independent in compartmental models. To compute the spatiotemporal distribution of drug concentration within each compartment, we integrated the compartmental model with the CDR equations [42]. A block diagram illustrating the compartmental model of IP drug delivery is presented in Figure 2.

### 2.1. Governing Equations

Drug delivery equations in tumor tissue are presented in this section as two different sets of equations: (i) interstitial fluid flow, and (ii) drug transport in tissue.

(i) Interstitial fluid flow

Given the intervascularity of the tumor interstitium, it is reasonable to assume that tumor tissue is a porous environment [43,44]. In this case, Darcy’s law is employed to characterize the tumor’s interstitial fluid flow as [30]

(1) $$v_{i}=-K\nabla P_{i}$$

In which *K* (m^2^/(Pa∙s)) is the hydraulic conductivity of the interstitium, ∇ represents the divergence operator, *P_i_* (Pa) is the IFP, and *v_i_* (m/s) stands for the IFV. *K* is frequently described as a function of tissue permeability, *k* (m^2^), and dynamic viscosity of the fluid, *μ* (Pa∙s), as

(2) $$K=\frac{k}{\mu }$$

For incompressible interstitial fluid in the interstitium, the steady state continuity equation is presented as [30]

(3) $$\nabla v_{i}=\phi_{B}-\phi_{L}$$

In which $\phi_{L}$ (s^−1^) and $\phi_{B}$ (s^−1^) represent the fluid flow from the interstitium to the lymph system and fluid flow from the microvascular system to the interstitium, respectively. It is assumed that there is not a functional lymph system in solid tumors, so $\phi_{L}=0$ in tumor tissue. However, $\phi_{B}$ term can be achieved through Starling’s equation as [42]

(4) $$\phi_{B}=\frac{L_{P}S}{V}\left(P_{B}-P_{i}-\sigma_{s}\left(\pi_{B}-\pi_{i}\right)\right)$$

In which *L_P_* (m/(Pa∙s)) is microvessels’ hydraulic conductivity, *S*/*V* (m^−1^) demonstrates the surface area per unit volume of microvessels, *P_B_* (Pa) is blood pressure, *σ_s_* represents osmotic reflection coefficient, *π_B_*_,_ and *π_i_* (Pa) stand for the osmotic pressure of microvessels and interstitium, respectively.

(ii) Drug Transport

The following equation can be used to obtain the free drug’s concentration in interstitium based on the CDR equation [25,39]:

(5) $$\frac{\partial C_{F}}{\partial t}=-v_{i}\nabla C_{F}+D_{F}\nabla^{2}C_{F}-\frac{1}{\varphi }K_{ON}C_{rec}C_{F}+K_{OFF}C_{B}+\Phi$$

where *C_F_* and *C_B_* (mol/m^3^) express the free and bound drug concentration in the interstitium, *C_rec_* stands for the cell surface receptors’ concentration, *K_ON_* and *K_OFF_* (1/s) are the association and dissociation rate of the drug agents to cell receptors, respectively. *D_F_* (m^2^⁄s) is the therapeutic agents’ diffusion coefficient and φ is the volume fraction of the tumor accessible to the drug. *Φ* is a source term demonstrating drug exchange among microvessels, interstitium, and lymph system, which can be derived as

(6) $$\Phi =\Phi_{B}-\Phi_{L}$$

*Φ_L_* represents the sink term for drug concentration caused by the lymphatic system, which is negligible because the tumor lacks a functional lymphatic system. Also, *Φ_B_* is the drug concentration source provided by microvessels [45,46]:

(7) $$\Phi_{B}=\left(\phi_{B}\left(1-\sigma_{f}\right)C_{P}+\frac{PS}{V}\left(C_{P}-C_{F}\right)\frac{Pe}{e^{Pe}-1}\right)$$

where *σ_f_*, *C_P_* (mol/m^3^), and *P* (m/s) are the filtration reflection coefficients for the drug, injected drug concentration into the microvessels, and vessel wall’s permeability. Peclet number, *Pe*, which reflects the ratio of mass transport via advection to drug transport along microvessel walls, is as follows:

(8) $$Pe=\frac{\phi_{B}(1-\sigma_{f})}{P\frac{S}{V}}$$

The concentrations of the drug that is bound and subsequently internalized are computed as follows [25]:

(9) $$\frac{\partial C_{B}}{\partial t}=\frac{1}{\varphi }K_{ON}C_{rec}C_{F}-K_{OFF}C_{B}{-K}_{INT}C_{B}$$

(10) $$\frac{\partial C_{I}}{\partial t}=K_{INT}C_{B}$$

where *C_B_* and *C_I_* represent bound and internalized drug concentrations. In addition, *K_INT_* is a constant that represents the internalization rate of the drug into cellular space.

The treatment efficacy, indicated by the fraction of killed cells (FK), is computed for Doxorubicin as follows [47]:

(11) $$FK=1-\exp\left(-\omega \cdot C_{I}\right)$$

In which *ω* is a fitting parameter that was obtained from the experiment [48].

### 2.2. Numerical Modeling

#### 2.2.1. Boundary Conditions

Since the time scale of tumor growth is higher than the treatment time scale, the boundary conditions are considered constant during the simulation. At the tumor surface, where the drug particles are in direct contact with the tumor, the drug concentration is considered to have a constant value (0.8 mol/m^3^). The constant pressure boundary condition for the inlet and outlet is considered equal to 25 mm Hg and 10 mm Hg, respectively. Additionally, a fixed zero-pressure boundary condition is established at the outer periphery of the tumor.

#### 2.2.2. Solution Strategy

**Figure 3:**
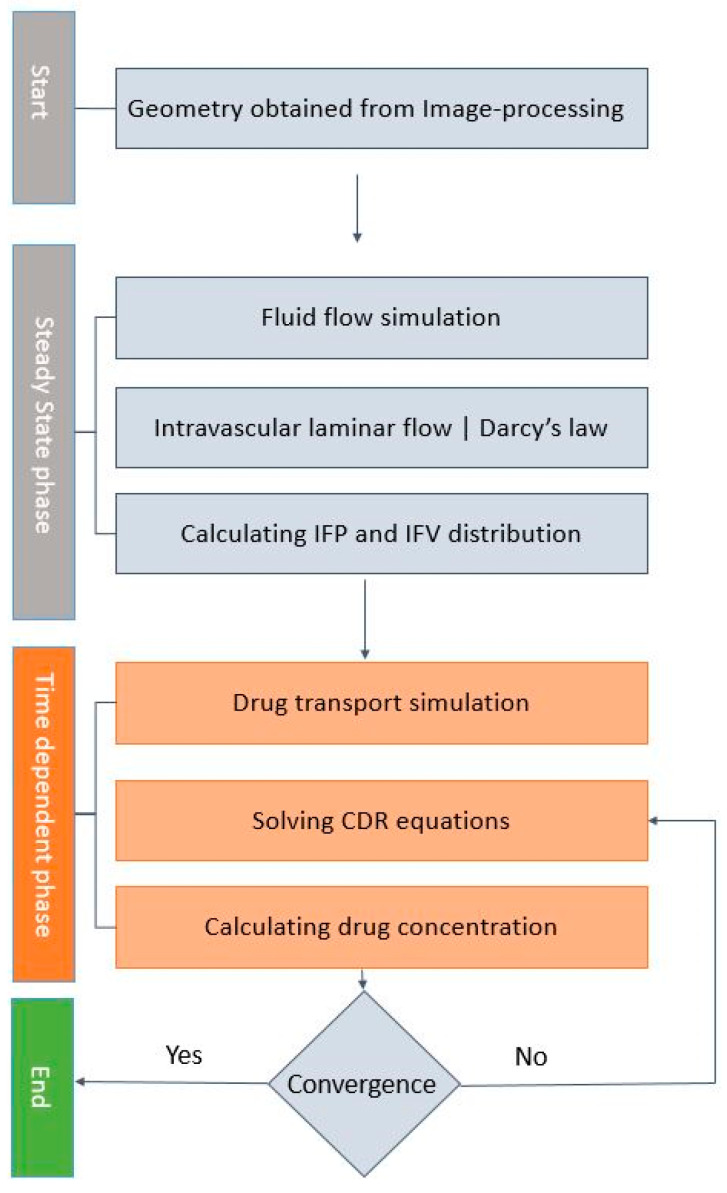
Flowchart describing the simulation process.

The flowchart describing the solution strategy of the simulation is shown in Figure 3. The solution process comprises two distinct phases: the steady state phase, which solves the intravascular and interstitial fluid flow equations, and the time-dependent phase, which solves the mass transport equations. The results of the steady-state solution were used as the input for transient simulations. According to the duration of the IP treatment method, the transient simulations were performed in one hour. As a convergence criterion, a 4-fold drop in magnitude in the residuals was chosen. The parameters of the model are summarized in Table 1.

#### 2.2.3. Model Geometry

**Figure 4:**
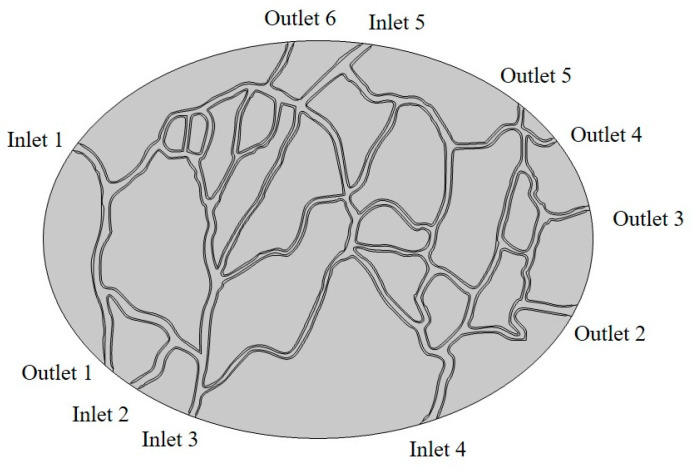
The geometry used in the simulation. There are 5 inputs named Inlets 1–5 and 6 outputs named Outlets 1–6 in the model.

In this model, representing the peritoneal tumor, an ellipsoid geometry is adopted with a long axis length of 4.85 mm and a short axis length of 3.51 mm (Figure 4). This geometry also includes a vascular network that is extracted from a real image by using image processing techniques. Section 2.2.4. describes the steps of converting the initial image to the final geometry used in the model. The model’s geometry includes 5 inlets and 6 outlets in the vessels, named Inlets 1–5 and Outlets 1–6 in Figure 4.

#### 2.2.4. Image Processing Method

**Figure 5:**
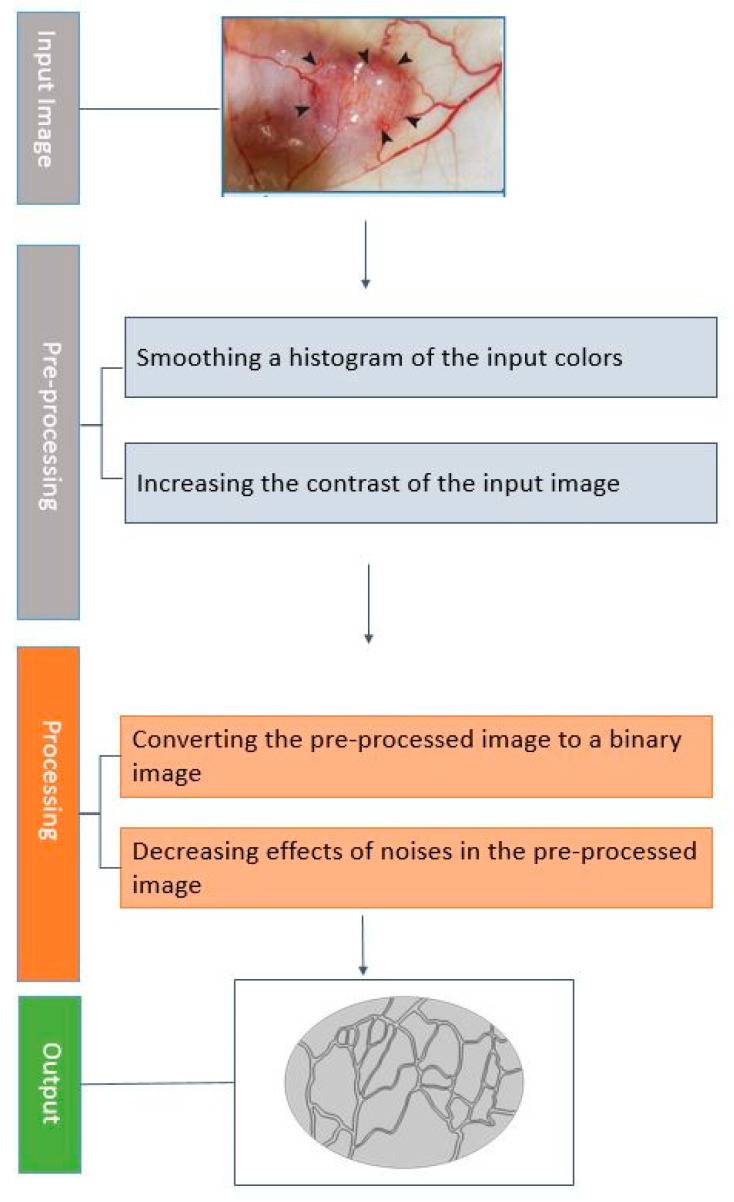
The image processing workflow in this study. The input image is extracted from [54].

In this study, we used a real tumor image with a capillary network, extracted from Roudnicky et al. [54], as input. Image processing is necessary for creating a standard geometry from an image containing capillaries. The aim is to accurately separate the capillary geometry from the background of the input image. By employing MATLAB software version R2023a as an image-processing tool, we initially generated a binary image. This was achieved by eliminating unnecessary background details from the input image through techniques such as histogram equalization for color intensity. Subsequently, following the assessment of the processed image contour, the minimum values are extracted to form a closed surface accurately representing the actual capillaries within the tumor. Figure 5 outlines the image processing workflow employed in this study on the input image.

#### 2.2.5. Grid Generation

**Figure 6:**
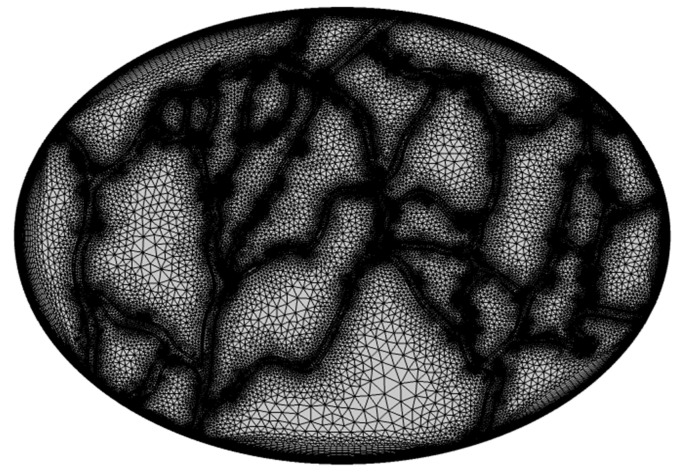
The final mesh generated for the model.

Since drug entry occurs from the outer boundary of the tumor, the spatial variations of the main investigated parameters, including IFP and concentrations, are very high in these areas. In addition, the area close to the tumor vessels is also important because of the fluid and mass exchange between tissue and vessels. For this reason, a boundary layer network has been used for the meshing of the geometry for the areas close to the outer edge of the tumor and the tumor vessels. This boundary layer contains 21 layers with the step of 1.2. For other areas of the geometry, the free triangular mesh has been used to achieve the desired accuracy in calculations. Figure 6 shows the generated mesh for the model. The total number of elements is 125,034, including 16,532 rectangular and 108,502 triangular elements.

## 3. Results and Discussion

In previous studies, IP drug delivery was modeled by considering tumor vessels as source terms [23,34,36,37,38,39]. While this approach to modeling the vascular network provides valuable insights into fluid and drug transport within tumors, research has highlighted the potential influence of tumor vasculature heterogeneity on drug distribution within the tumor [23,55]. In the present study, we have explicitly integrated the tumor’s vascular network into the model, utilizing a real image as a reference. To ensure a precise geometry for the tumor vascular network, image processing techniques were employed on the original real image of the tumor vasculature. Subsequently, utilizing this refined geometry alongside parameters related to the tumor tissue and the chemotherapy drug, we simulated the interstitial fluid flow and drug delivery within the tumor.

**Figure 7:**
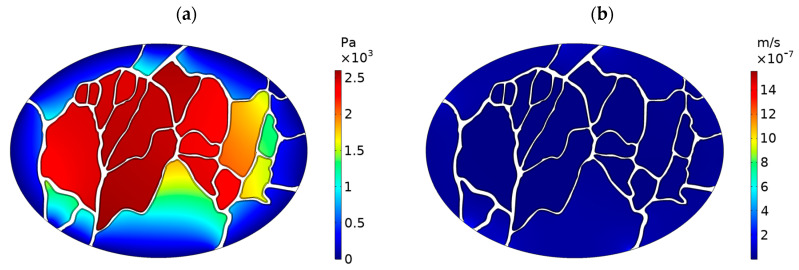
Distribution of (**a**) interstitial fluid pressure (IFP), and (**b**) interstitial fluid velocity (IFV) in the tumor.

Figure 7a shows the IFP distribution in the tumor. Notably, IFP reaches its peak in the central regions of the tumor, while it experiences a rapid decline towards the outer areas. This increased IFP value is in general agreement with the previous experimental and modeling studies [28,36]. By incorporating the tumor vessels into the model’s geometry, we observe a distinctively non-uniform pressure distribution within the tumor. This observation highlights that the heterogonous vasculature of the tumor directly contributes to the non-uniform distribution of IFP. Given that the IFV value is directly proportional to the gradient of IFP as defined in Equation (1), it follows that the IFV value experiences an increase in the outer regions of the tumor (Figure 7b).

**Figure 8:**
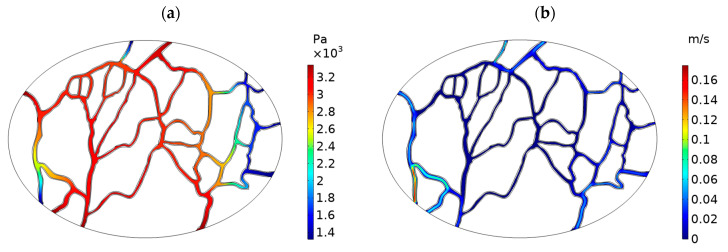
Distribution of (**a**) intravascular blood pressure (IBP), and (**b**) intravascular blood velocity (IBV) in the tumor.

Unlike chemotherapy with intravenous (IV) administration, in IP chemotherapy, the tumor vessels do not have the role of transporting the drug to the tumor tissue. However, the vascular network of the tumor is still influential in drug transport during IP chemotherapy [55]. Fluid exchange between blood vessels and tumor tissue determines the IFP and IFV, which is the basis for drug transport in tumor tissue [30]. In addition, the drug particles can be removed from the tumor tissue by intravasation into blood vessels [40]. Hence, intravascular blood pressure (IBP) and intravascular blood velocity (IBV) are important in determining fluid/mass exchange between blood vessels and tumor tissue. Figure 8a and b show the contours of IBP and IBV distribution, respectively. The ratio of minimum to maximum IBP in the simulation is 39.5, which is in agreement with the values obtained by [56,57]. In general, high IBP by strengthening the fluid convection to the interstitial space can increase the IFP values. This mechanism can ultimately hinder effective drug penetration into the tumor during IP chemotherapy.

**Figure 9:**
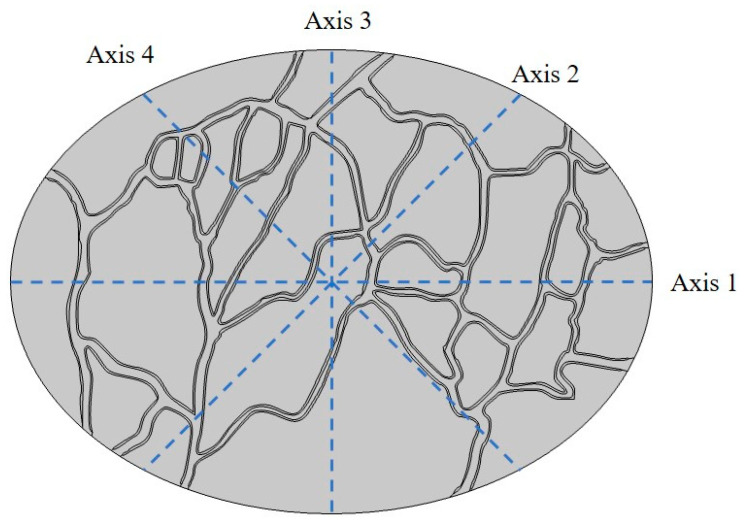
The positioning of various axes employed in the studies.

**Figure 10:**
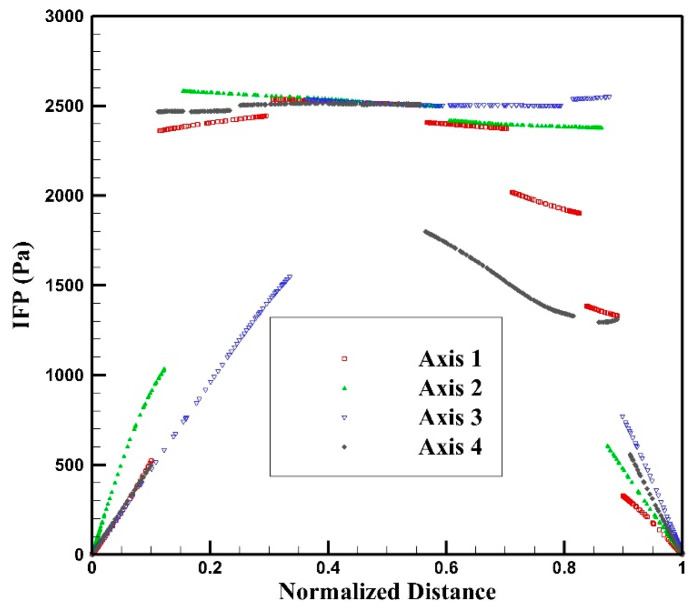
Interstitial fluid pressure distribution along axes 1–4.

To gain insights into the spatial distribution of interstitial fluid pressure/velocity and drug concentration within the tumor, four distinct axes have been positioned across the model geometry, as depicted in Figure 9. The distribution of IFP along axes 1 to 4 is illustrated in Figure 10. This figure demonstrates the presence of a heterogeneous IFP distribution within the tumor. The average IFP in the tumor is 1433.5 Pa, which is consistent with the values reported in previous numerical and experimental studies [28,36,58]. Furthermore, the IFP value within the central regions of the tumor has escalated to exceed 2500 Pa. The highest IFP value within the tumor reaches 2604.8 Pa. This value notably surpasses the maximum IFP in our earlier non-image-based model, which fell within the range of 1540 Pa. [39,42,59]. In general, a tumor typically exhibits elevated IFP at its center, attributed to factors such as a denser extracellular matrix, the lack of functional lymphatic vessels, and microvessels that display greater leakage compared to normal vessels. When contrasting the IFP profiles along axes 1 to 4, as depicted in Figure 10, it becomes evident that variations in IFP across different tumor regions are influenced by the distribution pattern of vessels within the tumor.

**Figure 11:**
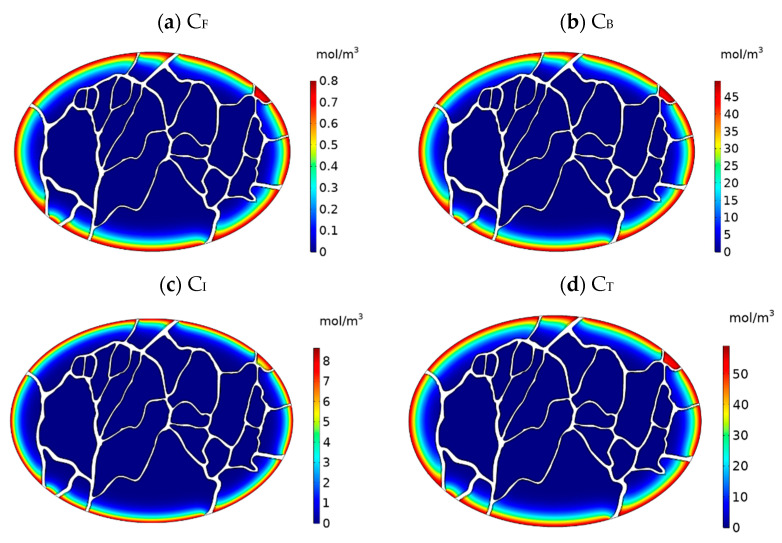
Contours of Doxorubicin concentration distribution in the tumor. (**a**) Concentration of free Doxorubicin C_F_, (**b**) concentration of bound Doxorubicin C_B_, (**c**) concentration of internalized Doxorubicin C_I_, and (**d**) total concentration of Doxorubicin C_T_.

**Figure 12:**
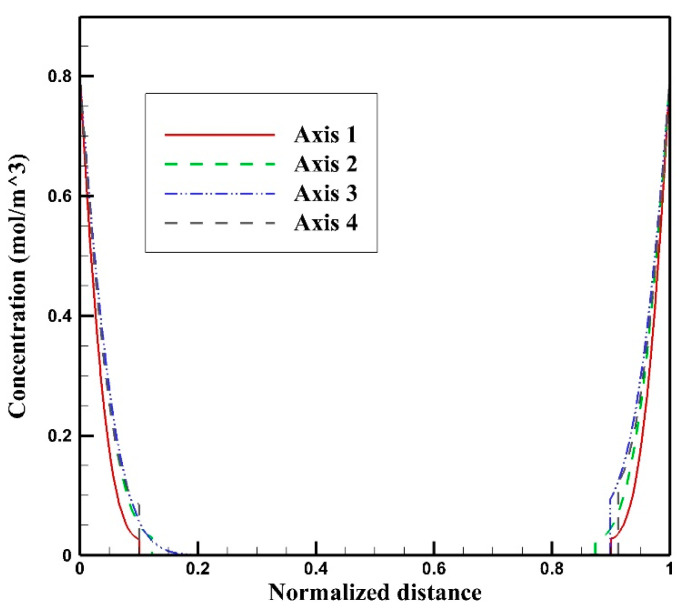
Free Doxorubicin concentration C_F_ distribution along axes 1–4, one hour after drug injection.

Figure 11a–d display the concentration contours of free (C_F_), bound (C_B_), and internalized (C_I_) Doxorubicin, along with the total Doxorubicin concentration (C_T_), within the tumor one hour after the initiation of drug injection. Doxorubicin’s penetration into the tumor primarily occurs in the outer regions, with a noticeable decline in concentration as one moves toward the tumor’s center. This restricted drug penetration is influenced by underlying factors associated with interstitial fluid flow within the tumor. The heightened IFV at the outer boundary triggers an outward convective flow, which consequently restricts the delivery of Doxorubicin to the tumor. Although the general trend aligns with what was observed in previous non-image-based models, a closer analysis of the concentration contours reveals that, unlike these earlier models, the distribution of drug concentration varies across different axes of the tumor. To facilitate a more comprehensive understanding, the concentration distribution profiles along axes 1 to 4 are presented in Figure 12. Notably, the extent of drug penetration varies distinctly along these axes. Consequently, in addition to being confined to the tumor’s outer boundary, drug penetration exhibits distinct patterns along different axes. To evaluate drug penetration within the tumor, we computed the half-width parameter W_1/2_, defined as the distance across the tumor surface where the free drug concentration C_F_ equals half of the exterior concentration (as described by Au et al. [34]). The half-width values, W_1/2_, ranged from 0.11 to 0.14 mm, varying along different axes within the tumor. This variance can be attributed to the heterogeneous distribution of vessels within the tumor, which subsequently leads to non-uniform distributions of IFP and IFV. As a natural consequence of this fluid flow heterogeneity, the distribution of drug concentration within the tumor is similarly influenced, thus amplifying the complexity of drug delivery.

**Figure 13:**
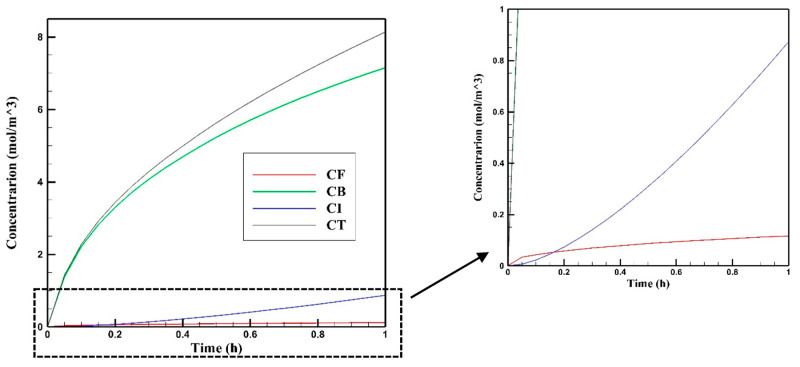
Mean Doxorubicin concentration in the tumor versus time for free, bound, internalized, and total Doxorubicin concentration during one hour of treatment with IP injection.

**Figure 14:**
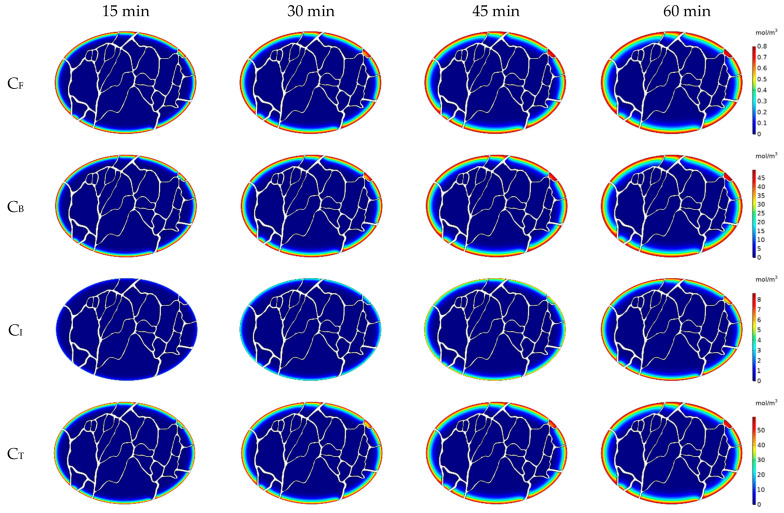
Contours of free, bound, internalized, and total Doxorubicin concentrations at 15, 30, 45, and 60 min after the start of treatment.

Figure 13 shows the average free, bound, internalized, and total drug concentration profiles in the tumor during 60 min of IP chemotherapy. With the start of injection, C_F_ increases from the initial value of zero. Upon entering the tumor, free Doxorubicin is moved deeper into the tissue through a combination of convection and diffusion mechanisms. Once within the tumor, it has the potential to bind to cancer cells. Through a continuous process of introducing free Doxorubicin into the tumor during injection, its conversion into bound drug, and subsequent internalization into cancer cells, the concentrations of bound (C_B_) and internalized (C_I_) Doxorubicin within the tumor escalate. Meanwhile, C_F_ experiences a more gradual increase, with a slope lower than that observed at the onset of injection. As depicted in Figure 13, it is notable that C_B_ consistently remains higher than the concentrations of the other two drug forms, namely C_F_ and C_I_. The interaction between the drug and the cells, characterized by the constants of drug binding, unbinding, and internalization rate, plays a pivotal role in governing the interconversion between these different drug forms. To visualize the spatiotemporal evolution of the drug delivery, concentration distribution contours are depicted at four distinct time intervals: 15, 30, 45, and 60 min, as illustrated in Figure 14. These contours effectively demonstrate the progressive increase in concentration over time, as well as the extent of drug penetration into the tumor.

**Figure 15:**
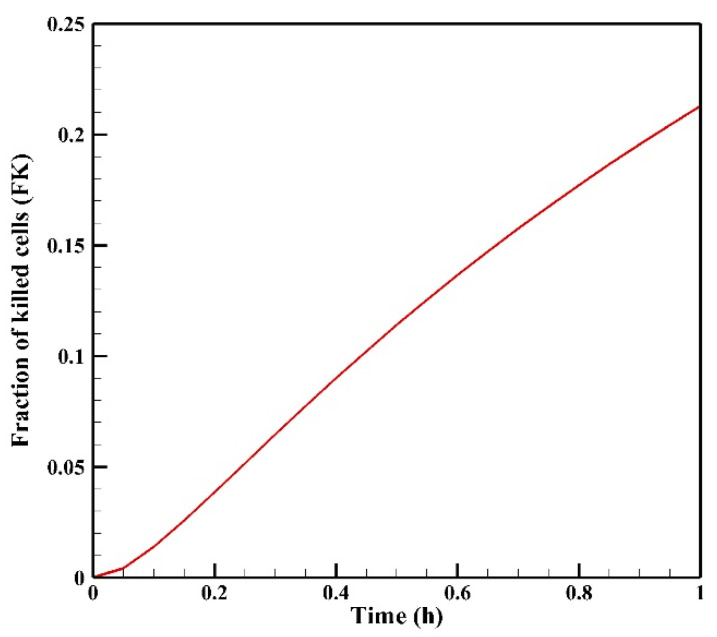
Fraction of killed cells (FK) versus time during one hour of IP chemotherapy.

To assess the level of drug exposure within the tumor, we calculate the area under the curve (AUC) of the free drug concentration (C_F_) versus time. The AUC value reached 295.18 mol·m^−3^·s after 60 min of treatment with Doxorubicin. Furthermore, for a quantitative assessment of treatment effectiveness, Figure 15 illustrates the fraction of killed cells (FK) versus time. As depicted, the FK value at the end of the 60-min treatment period stands at 0.204. It is worth noting that FK is directly influenced by the concentration of internalized drug (C_I_), leading to a gradual increase in FK with rising C_I_ levels within the tumor. Nevertheless, due to the constraints of drug transfer inherent in IP chemotherapy, the FK value remains relatively modest by the conclusion of the treatment.

## 4. Model Validation

We validated the results of our computational model through a comparison with previous experimental and numerical studies. Our model first solves the fluid flow equations, including intravascular and interstitial fluid flow, and then the drug transport equations. In this section, we have validated the results of both the fluid flow and mass transport solvers.

### 4.1. Validation of Fluid Flow Simulations

We initially validated our interstitial and intravascular fluid flow simulations by comparing them to previous studies. Our study recorded a maximum interstitial fluid velocity (IFV) value of 2.67 μm/s. This value falls well within the range of values reported in earlier experimental and modeling works [56,60]. Furthermore, our observed trend of increased interstitial fluid pressure (IFP) aligns with the general findings from previous experimental data, as demonstrated by Boucher et al. [28]. Moreover, the ratio of the minimum to maximum IBP in our simulation stands at 39.5, which is consistent with values obtained in other studies [56,57]. Additionally, the average IFP within the tumor, recorded at 1433.5 Pa, agrees with the values reported in previous numerical and experimental studies [28,36,58].

### 4.2. Validation of Mass Transport Simulations

**Figure 16:**
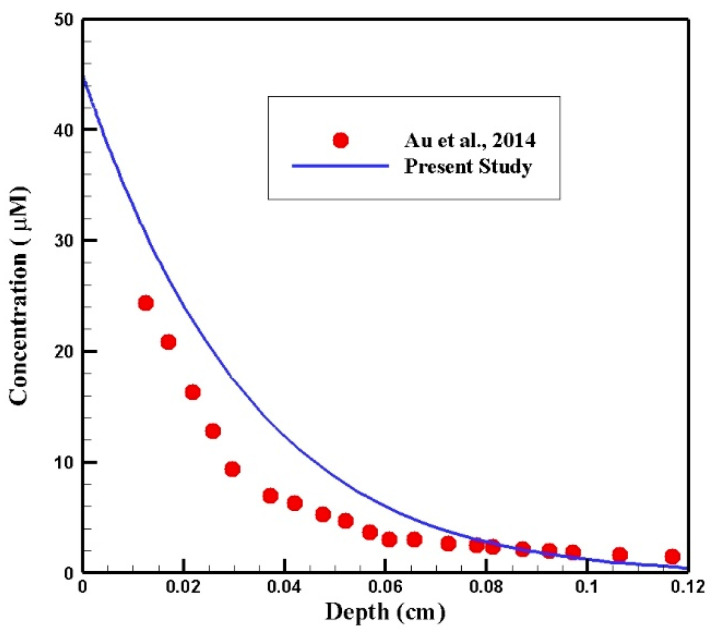
Validation of drug concentration distribution in the current study against the experimental data by Au et al. [34].

After confirming the performance of our fluid flow solver, we proceeded to validate the results of our mass transport model. We validated the results against experimental data presented by Au et al. [34], which detailed drug concentration profiles of drugs as a function of distance from the tumor periphery in mice after six hours of IP injection. Similarly, our simulation involved IP chemotherapy over a six-hour duration, setting the exterior concentration to 45 µM. A comparison between the results of our modeling and the experimental study by Au et al. is presented in Figure 16. Our results exhibit similar trends to the experimental data. However, differences between our simulation and the experimental data by Au et al. [34] are observed. These can be attributed to variations in drug properties and tumor tissue parameters between the two studies.

## 5. Limitations and Future Work

In our study, we have certain assumptions and limitations that merit discussion. First, the drug concentration at the outer edge of the tumor was held constant throughout the simulation, primarily due to the unavailability of experimental data. Addressing this limitation by implementing more realistic boundary conditions for concentration at the tumor’s outer edge would undoubtedly enhance the model’s accuracy in future works. Another limitation to consider is our utilization of a two-dimensional tumor model instead of a more comprehensive three-dimensional representation. While this approach allowed us to explore the initial facets of our research, it is important to acknowledge that a full three-dimensional model will better represent real-world situations. Furthermore, our choice to employ a static network for capillaries in our model represents a simplification of the dynamic nature of microvascular networks in vivo. Similarly, assuming laminar flow for blood circulation within the microvascular network is another simplification that may not fully capture the complexity of blood flow patterns. Lastly, we assumed uniform transport properties within the tumor tissue. In reality, these properties can exhibit heterogeneity, which may impact the accuracy of our model’s predictions. It is important to recognize these limitations as they guide opportunities for future research aimed at refining and expanding our understanding of the subject matter.

## 6. Conclusions

Chemotherapy by injection into the peritoneal cavity is a promising method for the treatment of tumors in the peritoneal region. Even though IP chemotherapy provides a locoregional therapy that delivers more drug particles to the tumor compared to IV chemotherapy with the same systematic side effects, drug penetration in this method is limited. Drug transfer in a tumor is affected by various parameters of tumor tissue, the chemotherapy drug, and the administration route. Considering the tumor as a porous medium, the interstitial fluid of the tumor is the medium for the transport of drug particles. Therefore, the interstitial fluid flow in terms of IFP and IFV has a major impact on the drug delivery in tumors. The interaction between the vascular network and the interstitial space of the tumor leads to fluid/mass exchange between these two compartments. Therefore, tumor vessels are potentially influential on the interstitial fluid flow and drug delivery in tumors. In this study, a mathematical model based on diffusion–convection–reaction equations is presented to investigate IP drug transfer in a tumor with a heterogeneous vasculature. In the previous models, the role of tumor vessels was considered as source/sink terms. In other words, it was assumed that the vessels are homogeneously distributed in the tumor. This was a simplifying assumption because, in reality, there are tumors with different vascular networks that often have a heterogeneous distribution. The model presented here, unlike previous models that used a simplified ideal tumor geometry, is based on a real image of the vascular network, and the influence of the vessels is seen in it. The results showed how the vascular network of a tumor is effective in drug delivery during IP chemotherapy. The key findings of this study can be summarized as follows:

1. The tumor’s vascular network, characterized by its heterogeneous distribution of vessels, contributes to heterogeneous distributions of interstitial fluid pressure (IFP) and interstitial fluid velocity (IFV) within tumor.
2. Drug penetration within the tumor exhibits diverse patterns along different axes in the tumor as a consequence of the heterogeneous distribution of vessels and fluid flow in the tumor, thus increasing the complexity of drug delivery.
3. The geometric attributes and unique vascular network of tumors are crucial considerations before treatment planning.

The image-based model presented here signifies the initial strides toward personalized medicine for treating patients with peritoneal carcinomatosis. It offers unique insights into the various challenges encountered during IP chemotherapy. Additionally, this model lays the foundation for developing a computational tool capable of assessing and predicting IP chemotherapy outcomes based on patient-specific data. Furthermore, when compared to in vivo studies, this model presents a cost-effective alternative for testing various drugs and treatment protocols, aiding in the identification of novel personalized treatment strategies for patients. The results generated by this model also contribute valuable insights to the drug transport processes during IP chemotherapy, with the potential to benefit future preclinical investigations.

## Figures and Tables

**Table 1:** Parameters used in the numerical model.

Parameter	Definition	Unit	Value	Reference
*S*/*V*	The surface area of blood vessels per unit of tissue volume	m^−1^	2 × 10^4^	[49]
k	Hydraulic conductivity of the interstitium	m^2^·Pa^−1^·s^−1^	3 × 10^−14^	[50]
*L_P_*	Hydraulic conductivity of the micro-vascular wall	m·Pa^−1^·s^−1^	2.10 × 10^−11^	[50]
*P_B_*	Vascular fluid pressure	Pa	2.1 × 10^3^	[50]
*π_B_*	The osmotic pressure of the plasma	Pa	2.7 × 10^3^	[50]
*π_i_*	The osmotic pressure of the interstitial fluid	Pa	2 × 10^3^	[50]
*σ_s_*	Average osmotic reflection coefficient for plasma proteins	-	0.9	[50]
*D_eff_*	Effective diffusion coefficient	cm^2^·s^−1^	3.40 × 10^−6^	[49,51]
*P*	Microvessel permeability coefficient	cm·s^−1^	3.00 × 10^−4^	[49,51]
*K_ON_*	Constant of binding rate	M^−1^·s^−1^	1.5 × 10^2^	[25,52]
*K_OFF_*	Constant of unbinding rate	s^−1^	8 × 10^−3^	[25,52]
*K_INT_*	Constant of cell uptake rate	s^−1^	5 × 10^−5^	[25,52]
*φ*	Tumor volume fraction accessible to drugs	-	0.3	[53]
*C_rec_*	Concentration of cell surface receptors	M	10^−5^	[25]
*ω*	Cancer cell survival constant	m^3^·mol^−1^	0.6603	[47]

## Data Availability

All datasets and computational codes presented in this study are available on reasonable request from the corresponding author.
